# Toothbrushing behavior in children – an observational study of toothbrushing performance in 12 year olds

**DOI:** 10.1186/s12903-019-0755-z

**Published:** 2019-04-29

**Authors:** Renate Deinzer, Oliver Cordes, Julia Weber, Lisa Hassebrauck, Ulrike Weik, Norbert Krämer, Klaus Pieper, Jutta Margraf-Stiksrud

**Affiliations:** 1 Institute of Medical Psychology, Department of Medicine, Justus-Liebig-University Giessen, Klinikstr. 29, D-35392 Giessen, Germany; 2 Department of Medicine, Philipps University of Marburg, Georg-Voigt-Straße 3, D-35039 Marburg, Germany; 3 Department of Psychology, Philipps University of Marburg, Gutenbergstr. 18, D-35032 Marburg, Germany

**Keywords:** Oral hygiene, Community dentistry, Dental education, Dental hygiene, Preventive dentistry, Behavioral science, Toothbrushing

## Abstract

**Background:**

Many countries offer systematic group prevention programs in kindergarten and school in order to promote children’s oral health. Little is known, however, about the actual toothbrushing abilities of children when group prevention programs end.

**Methods:**

In Germany, all children take advantage from a nationwide group prevention program (called “Gruppenprophylaxe”) lasting from kindergarten up to sixth grade (12 years of age). Standardized recommendations are given concerning brushing systematics and brushing movements. *N* = 174 children at the age of 12 were thus randomly selected from two German towns and were asked to perform toothbrushing to the best of their abilities in front of a mirror which also served as a camera. Brushing behavior was analyzed by video analysis.

**Results:**

Children brushed their teeth for an average of 200 s ± 80.48 s (mean ± SD). Still, more than 55% missed at least one sextant when brushing inner surfaces, 16% missed them all. Only 7.5% of the children brushed both inner and outer surfaces by the intended movements (vertical movements on the inner surfaces and circular movements on the outer surfaces) for at least 90% of the respective brushing time. Instead, horizontal brushing was very common on the lateral surfaces.

**Conclusions:**

The present analysis indicates that children have low efficiency to adopt the tooth-brushing recommendations given in prevention programs. This is surprising as great endeavors are made to help children internalize the recommendations. Future research is needed to better understand which factors impede adoption of toothbrushing recommendations in children and which efforts are necessary to improve their toothbrushing abilities.

**Electronic supplementary material:**

The online version of this article (10.1186/s12903-019-0755-z) contains supplementary material, which is available to authorized users.

## Background

Plaque removal on a regular basis is a central part of oral health prophylaxis. Without proper oral hygiene (including fluoride toothpaste) there is a high risk for caries and gingivitis [1–4]. Accordingly, there is wide consent that oral hygiene behavior is essential for everyone and should begin with the first tooth of a child [5, 6]. As this health behavior has to be performed on a daily basis [6], it is important to educate children to take up the responsibility for their own oral health. Bad oral health can have extensive and unpleasant consequences for the child esp. for medically compromised children [7]. Toothaches, dental treatments and loss of the integrity of single teeth or even the dentition can be the direct consequences [8, 9]. Social rejection can be a further one when the child is teased or socially excluded because of visible defects, impeded articulation, or bad odors [10–12]. Thus proper oral health education not only helps to maintain the teeth and gingiva healthy but also prevents children from unpleasant somatic, psychological, and social experiences [13]. Still, one must not rely completely on the parents’ abilities to provide such education without any help. Deficits in oral hygiene skills and knowledge of adults [14–19] and a strong social gradient regarding oral health [20, 21] indicate that the society should take on responsibility here, too. Thereby, prevention programs have been developed in several countries [22, 23].

These programs aim to enhance oral health in children and enable them to maintain oral health when they reach adulthood. They rely on early and systematic approaches in kindergarten and school settings. Besides general nutrition information and getting accustomed to dental examination and dental staff, main efforts are invested in teaching tooth brushing techniques [22]. In Germany, this form of group prevention programs starts from the kindergarten and ends usually by the 6th grade when children reach the age of about 12 years [24, 25]. Group prevention programs contain clear instructions for the way in which the children should brush their teeth, based upon the cognitive and psychomotor abilities of the children as well as upon dental health purposes.

While there exist some evaluations of these programs which focus caries prevalence [20, 22, 23, 26–29] nothing is known about the tooth brushing abilities children have acquired at the end of group prevention programs. A direct evaluation of the results of tooth brushing instructions and training in terms of the intended elements of the brushing procedure is missing. Since proper tooth brushing is the most important for current oral health and the ability to maintain oral health in future, it appears to be worthwhile to evaluate these abilities.

The aim of the present study therefore was to examine whether children who are 12 years of age performed toothbrushing as per the given instructions by the end of the group prevention programs.

## Methods

This study conforms to STROBE Guidelines.

### Participants

In order to enhance the generalizability of results, recruitment of children took place in two small towns in Hesse, Germany (Marburg, approx. 73.000 inhabitants and Giessen, approx. 85.000 inhabitants), each having its own responsibility in application of group prophylaxis and each applying slightly different teaching methods. The assessments took place in dental examination rooms of the Institute for Medical Psychology, Justus-Liebig-University Giessen, and in the rooms of the Dental Department of the University of Marburg, respectively, from 2014 to 2015.

Inclusion criteria were: 1) born in 2003, 2) being a resident of Giessen or Marburg, 3) written informed consent of the child, 4) written informed consent of the parents. Exclusion criteria were: 1) fixed orthodontic appliances; 2) cognitive or physical impairment that affects toothbrushing; 3) habitual use of a powered toothbrush; 4) removable dentures.

A random sample from a list of all inhabitants born in 2003 and living in the respective town was drawn by means of a computer-generated list of random numbers and was invited via mail for participation. A final sample size of *n* = 100 per town was pursued. In case children did not respond to the invitation, denied participation actively or fulfilled the exclusion criteria the next on the list of random numbers was invited. A flow diagram of recruitment is shown in the Additional file 1.

### Toothbrushing instructions during group prevention

In Germany, due to social legislation all children receive group prevention from kindergarten up to the 6th grade [24, 25]. The staff providing the group prevention program (dentists and their assistants) usually visit groups at least once a year. As in many other regions in Hesse and Germany they use a supporting song which should help to explain the toothbrushing procedure and to accustom children to a fixed sequence of surfaces to be brushed and of movements to be applied. Furthermore, as it is available online [30] it allows other instructors like teachers, parents etc. to apply the same method. All these measures may help children to adopt a reasonable toothbrushing habit [31]. Another important advantage of this method is its high degree of standardization, which allowed for the formulation of concrete hypotheses about the brushing behavior if children follow the instructions. The hypotheses of the present research are based on the following details of the song: The full length of the song is 3:28 min. The time by which the child effectively brushes his/her teeth during the song (tooth contact time, see also below) is approximately 100 s. The rest of the time is used for further explanations how to change the position of the brush, where to start, etc. It is explicitly recommended to repeat the song if considered necessary by instructors, teachers or parents. The song consists of three verses. Each verse begins with some bars allowing the children to change the position of the brush. The first verse then instructs children to brush their occlusal surfaces (directly translated text: “Back and forth, back and forth, brushing teeth is not so hard”). This refrain is repeated four times, once for each quadrant. In between the child is instructed to change the position of the brush from the lower to the upper mandible. The next verse asks the children to close the mandibles (“like a tiger”). Then the refrain instructing them to brush the outer (vestibular) surfaces by circular movements is repeated three times once for each pair of antagonistic sextants (1–6, and) (translated text: “All around, all around, brushing teeth is healthy”). The last verse instructs them to brush their inner (palatinal) surfaces by vertical movements (directly translated text: “Wipe out, wipe out, wipe all the dirt out”). This refrain is repeated six times, once for each sextant. Thus, a child following these instructions brushes its inner surfaces twice as long as the outer surfaces and 1.5 times as long as occlusal surfaces. Within inner and outer surfaces one would expect it to brush sextants with equal length.

### General design

After arrival in the lab, children were asked to brush their teeth with the given toothbrush and tooth paste and were simultaneously recorded on a tablet computer with a front camera (also a mirror) adjusted to their height. They were asked to clean their teeth to the best of their abilities and were left alone while performing oral hygiene. Some questionnaires were also assessed, as were some clinical data. Being of no relevance for the current research question, they were not explored further. Only the DMFT (decayed, missing, filled teeth)-Index [32] is reported in order to allow the comparison of the oral health of the current group with representative samples. As a measure of socioeconomic status, the highest degree of education of the participants’ parents was assessed and dichotomized for later analyses (university entrance diploma or not).

### Observed oral hygiene behavior

The videos were analyzed by three independent calibrated examiners (authors OC and LH and assistant GAF) using the software Mangold INTERACT® 14 (Mangold International GmbH, Arnstorf, Germany). The examiners watched the video multiple times (also in slow motion) in order to code different behavioral categories. Calibration was provided by 10 videos of individuals that were not involved in the present study. The calibration criterion was an intraclass correlation of ICC > 0.80.

LH first coded total tooth contact time (time while toothbrush touches the teeth, without rinsing, spitting, tongue cleaning or breaks) and tooth contact time with respect to surfaces (inner, outer and occlusal). Next, brushing on the lateral surfaces was coded by an associate with respect to brushing movements (horizontal (scrubbing), vertical, circular, resembling the modified Bass-technique (jiggling and wiping out)) and by OC with respect to sextants (sextant 1 to 6, and concurrent brushing of outer surfaces of antagonistic sextants, i.e. 1 and 6, 2 and 5, 3 and 4, respectively). Concurrent brushing of antagonistic sextants was coded when participants closed the mandibles while brushing. For further analyses, the brushing time of two sextants brushed concurrently was distributed to equal parts to both sextants. No further rating of brushing on the occlusal surfaces took place. This was due mainly to the fact that scrubbing was the only brushing movement seen on the occlusal surfaces and that the visual differentiation of sextants on the occlusal surfaces is difficult. None of the examiners knew the clinical data of the participants at the time of coding. While OC did some of the clinical assessments, he analyzed videos weeks to months later. This should have diminished recognition of the respective case.

To ensure that coding remained reliable over time during the process of analyses, one video from each of 10 successive ratings was randomly chosen for double coding by another person. The person who did the main coding (coding of all videos regarding one behavioral aspect) was blinded with respect to the videos chosen for double coding and the person who did the double coding did not know the coding of the other rater. The concordance of these double ratings was always above ICC = .926 (intra class coefficient).

### Statistics

Descriptive and inferential statistics were computed by IBM SPSS Statistics 24 (IBM Corporation, U.S.A). Statistical significance was considered with *p* ≤ 0.05. Sample size allowed for the detection of small effect sizes with a power of 1-β = 0.95. According to the instructions during group prevention programs one would expect children to brush their inner surfaces twice as long as the outer surfaces, and 1.5 times as long as occlusal surfaces. To test whether the actual distribution of brushing time deviates from this assumption the brushing time of the surfaces was converted. The brushing time of outer surfaces was multiplied by the factor 2, that for occlusal surfaces was multiplied by 1.5 and brushing time of inner surfaces was multiplied by 1 (i.e. remained the same). This should have resulted in equal values of the converted variables. A repeated measures analysis of variance (ANOVA) tested for deviations from this assumption. According to toothbrushing instructions, one would further expect children to distribute brushing time equally to sextants both within inner and within outer surfaces. To test whether distributions of brushing time deviated from this assumption, two additional repeated measures ANOVAS were computed, one for inner and one for outer surfaces. Greenhouse Geisser’s ε was applied to all repeated measures ANOVAs to correct for violations of the sphericity assumption. Original degrees of freedom are reported together with Greenhouse Geisser’s ε. Due to apparent neglect of inner surfaces, a further descriptive analysis was run examining how many sextants were brushed for less than 1 s at inner surfaces and thus practically neglected. Finally, according to brushing instructions, one would expect children to brush inner surfaces mainly by vertical movements. To test this hypothesis, two Student t-tests for dependent measures were run, one comparing the time children brushed inner surfaces with vertical vs. circular movements and one comparing brushing time with vertical vs. horizontal movements. The hypothesis was to be accepted when both tests were significant. Thus, no correction for α-error-accumulation was necessary. Similarly, to test the hypothesis that outer surfaces were mainly brushed by circular movements, two t-tests were run to compare circular to vertical and circular to horizontal brushing, respectively. Additional descriptive statistics were computed in order to further explore the distribution of the extent by which children followed the instructions: a) percentage of time by which children closed their mandibles while brushing outer surfaces; b) percentage of time by which they brushed outer surfaces by circular movements, and inner surfaces by vertical movements; c) percentage of children who brushed their inner surfaces longer than their outer surfaces. Furthermore, as many children missed at least one sextant while brushing their inner surfaces, the number of sextants brushed less than 1 s was computed to further describe the extent of the neglect.

## Results

**Fig. 1:**
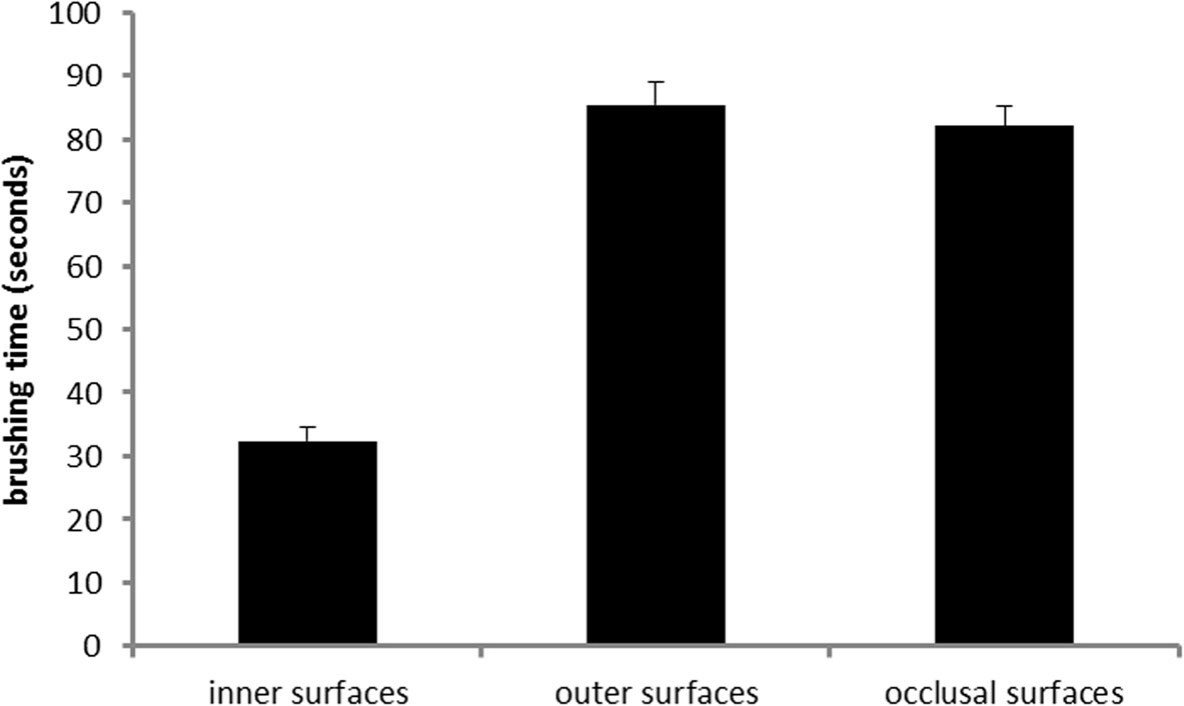
Means and standard error of the means of the duration of brushing of inner, outer and occlusal surfaces, respectively

**Fig. 2:**
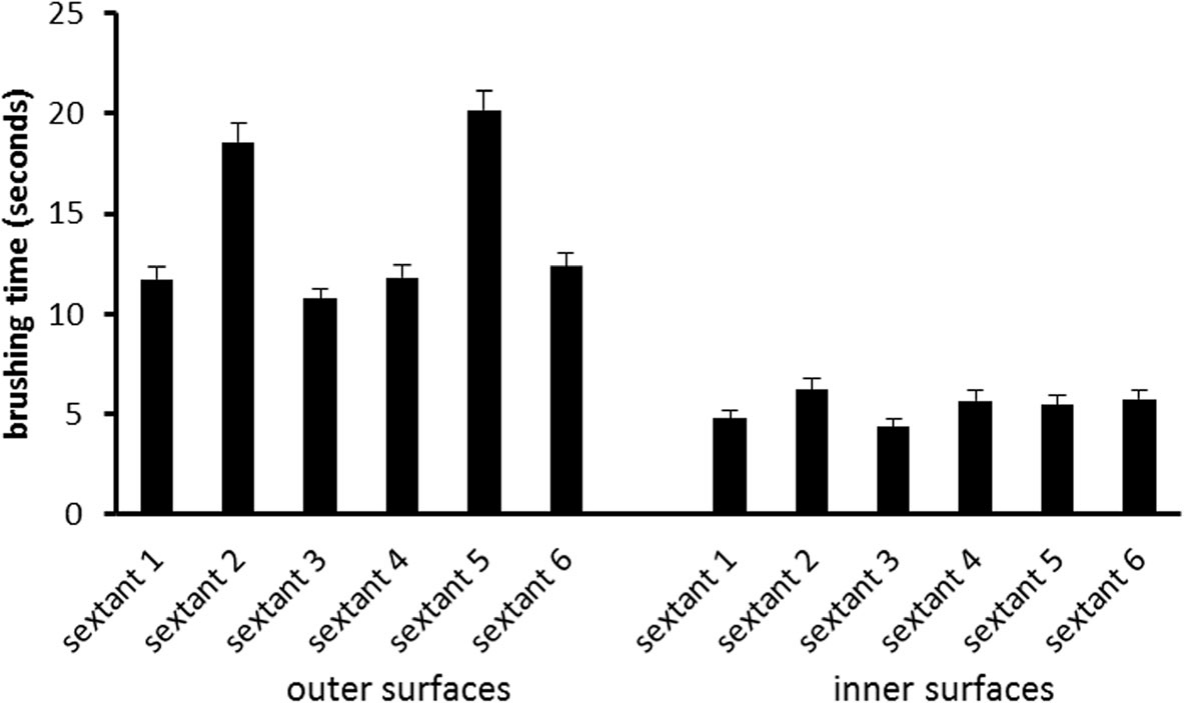
Means and standard error of the means of the duration of brushing of the respective sextants at outer and inner surfaces, respectively

**Fig. 3:**
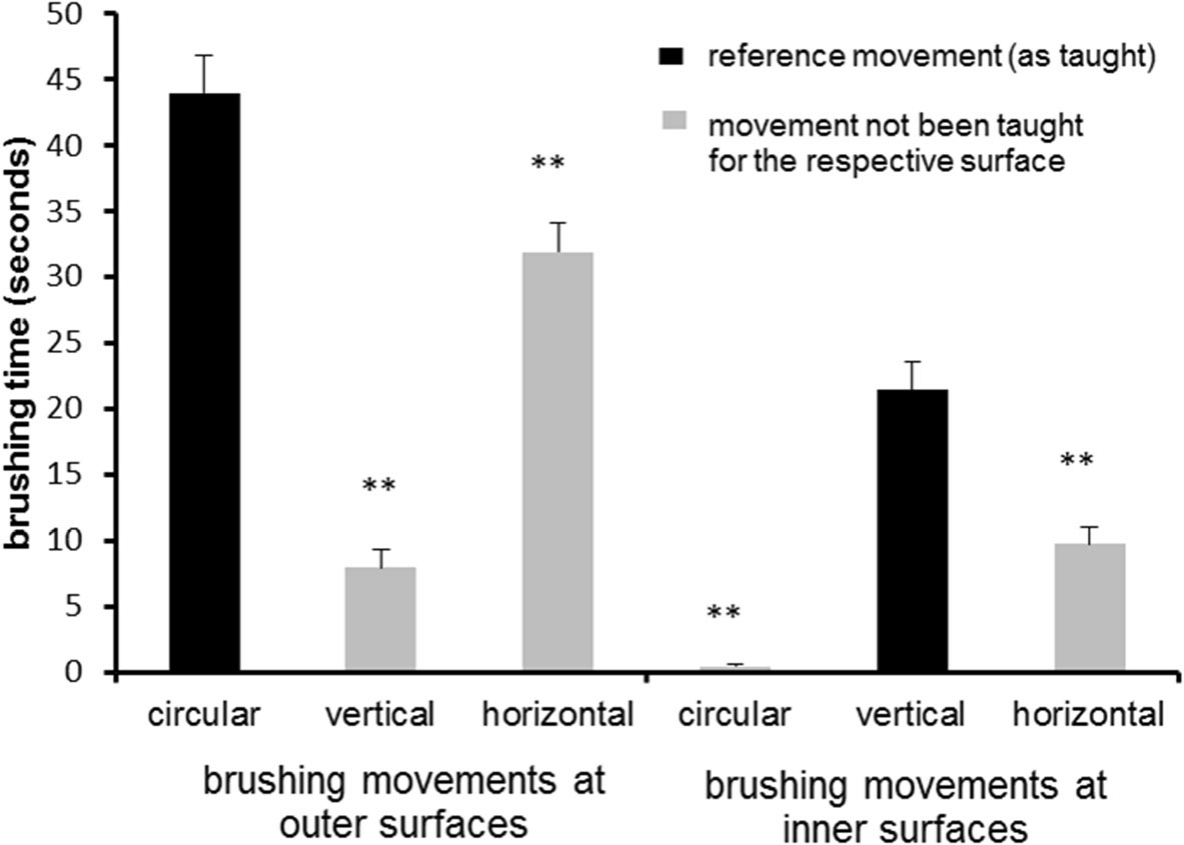
means and standard error of the means of the duration of the respective movements at outer and inner surfaces, respectively. **significantly (*p* ≤ .001) different from reference movement

From the original study sample (*n* = 189 children) *n* = 15 (six children of Marburg, 9 children of Gießen) were excluded from analyses as they were missing in the video for more than 5% of the total duration of brushing time. Table 1 shows the characteristics of the remaining children. *N* = 122 (70%) had no decayed or filled teeth and *N* = 21 (12%) had more than one decayed or filled tooth. *N* = 143 (82%) had at least one of their teeth sealed. They brushed their teeth by an average of 199.84 (± 80.48) seconds. Figures 1 and 2 shows the distribution of brushing time across surfaces (inner, outer, occlusal) and sextants by surfaces, respectively. All three ANOVAs revealed highly significant deviations from the assumptions of the null-hypothesis with large effect sizes. This indicates that children neither distributed brushing time as expected to surfaces (F(2/346) = 224.1; *p* < .001; ε = .842; η^2^ = 0.564) nor to inner (F(5/865) = 4.67; *p* = .001; ε = .844; η^2^ = .026) nor to outer (F(5/865) = 65.93; p < .001; ε = .419; η^2^ = .276) sextants. Regarding site specific application of brushing movements Fig. 3 indicates that inner surfaces were brushed significantly longer by vertical movements than by circular (t(173) = 10.055; p < .001) and horizontal (t(173) = 4.522; p < .001) movements, respectively. Outer surfaces were significantly longer brushed by circular than by vertical (t(173) = 11.202; p < .001) and horizontal (t(173) = 3.013; p = .001) movements, respectively.

**Table 1:** Characteristics of the sample

Town (n)	
Giessen: 90	
Marburg: 84	
Sex (n)	
male: 91	
female: 83	
educational status of parents (n):	
UED: 123	
no UED: 44	
unknown: 7	
brushing hand (n)	
right: 148	
left: 12	
both: 14	
Dental status (mean ± SD)	
permanent teeth: 22.7 ± 4.7	
DMFT: .57 ± 1.2	

UED: at least one parent with university entrance diploma

**Fig. 4:**
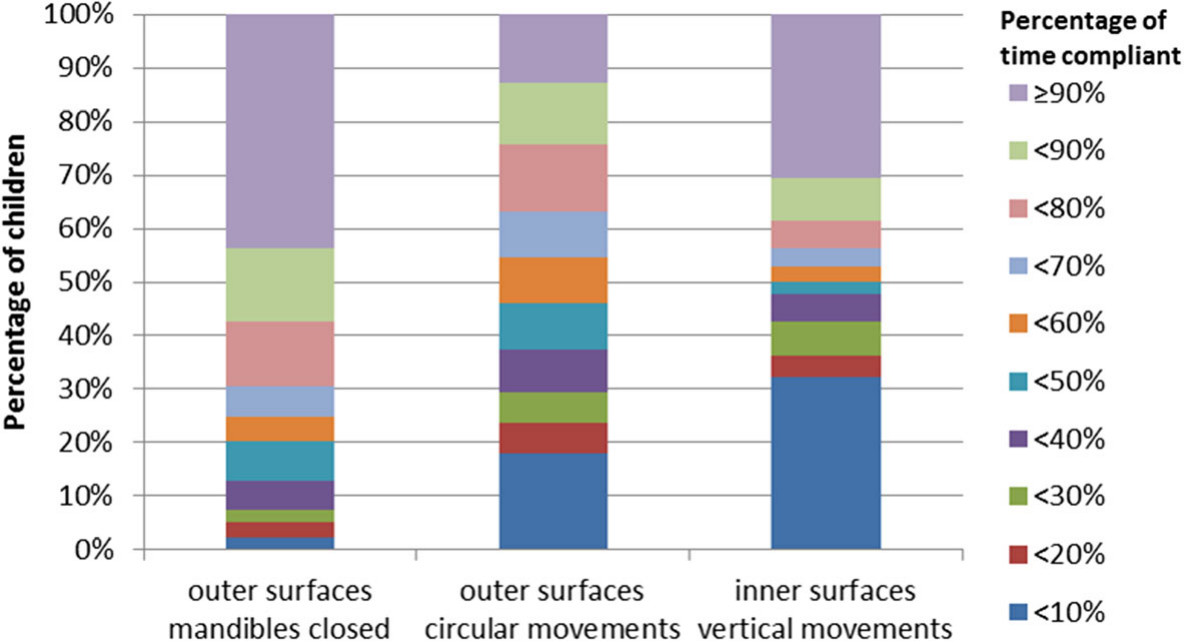
Distribution of the percentage of time by which children adhered to the respective brushing recommendations given in the brushing song

**Fig. 5:**
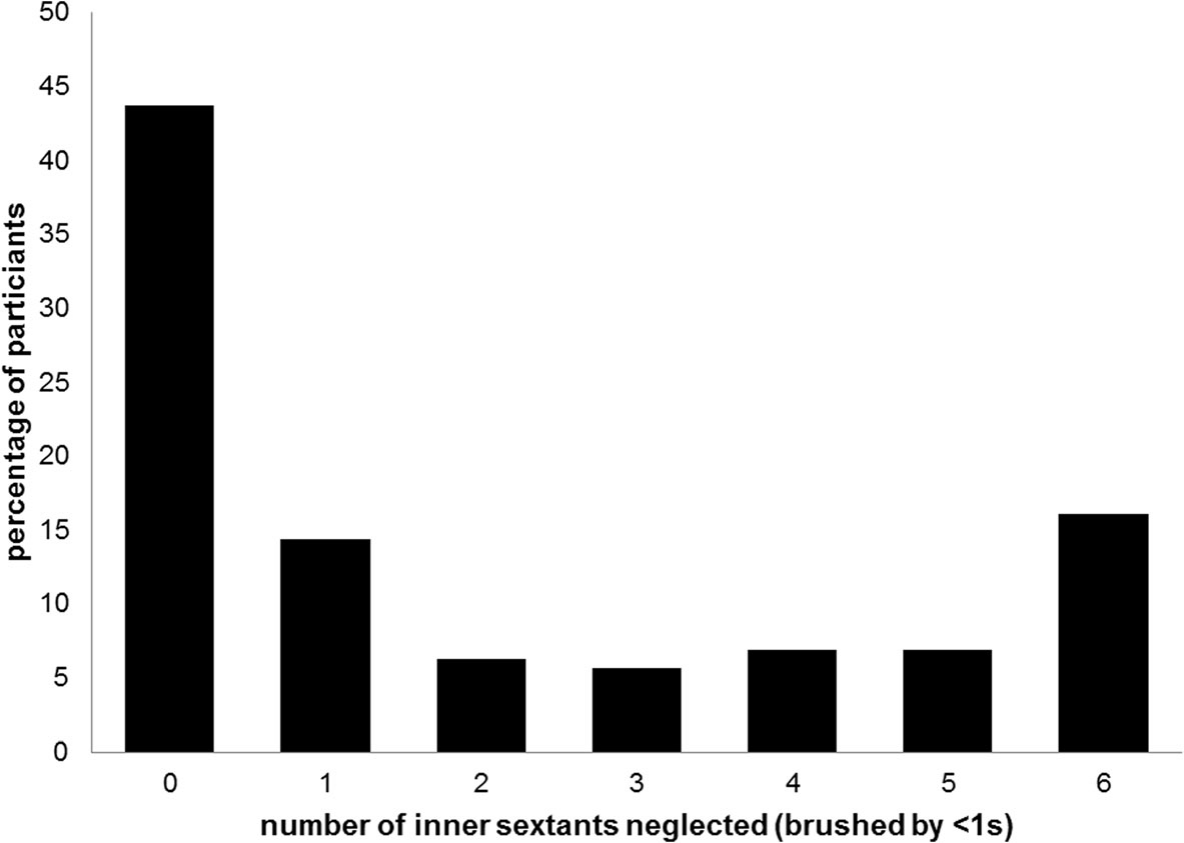
Percentage of children neglecting brushing of inner surfaces of 0–6 sextants

Figure 4 shows the distribution of the extent of compliance with respect to brushing recommendations. Thirteen children (7.5%) brushed both inner and outer surfaces for at least 90% of the respective brushing time by the intended movements. Seventeen children (9.8%) brushed their inner surfaces longer than their outer surfaces and 43.7% (*n* = 76) of the children managed to brush all inner sextants by at least 1 s while the remaining children missed at least one sextant (see Fig. 5). Regarding outer surfaces, all children brushed all sextants for at least 1 s (data not shown).

## Discussion

The present study analyzed toothbrushing behavior of 12-year-old children living in two German towns. Compared to the Fifth German Oral Health Study (DMS V [33];), the current sample appears to be well comparable to the German average of 12 year olds regarding DMFT (overall German mean: 0.5; current sample: 0.6) and percentage of children with at least one tooth sealed (overall German percentage: 70%; current sample: 82%). Thus, it is unlikely that specific health characteristics of the sample distort the results of the study.

Group prevention programs for oral health in Germany end at the age of 12. An important part of these programs is the instruction for brushing teeth. The children are taught specific brushing movements, which they should apply depending on the surface. Instructions also result in differential lengths by which inner, outer and occlusal surfaces should be brushed. The longest time is dedicated to inner surfaces and the shortest to outer surfaces. Additionally, within inner and outer surfaces, respectively, instructions distribute brushing time equally to sextants. The present study analyzed whether the actual brushing behavior of children at the age of 12 corresponded to the characteristics of the instructions they had received during group prevention programs. The results of the present study show a mixed picture.

First, circular, vertical and horizontal movements were actually more common in the intended regions: Vertical movements were rarely seen on the outer surfaces, as were circular movements on the inner surfaces (see Fig. 3). Still, children showed a considerable amount of horizontal movements at these surfaces (see Fig. 3). This is remarkable, as instructions definitely do not recommend these movements at lateral surfaces. However, the children should have brushed their occlusal surfaces by horizontal movements (which all of them did). As the sequence of brushing starts with the occlusal surfaces, one could speculate that children have difficulties to inhibit this movement pattern while going on to the other surfaces. Another explanation could be that horizontal movements are easier to perform than circular movements or vertical strokes and that children thereby tend to fall back into that behavior [34–38]. The results regarding the extent of adherence to the respective movements support these considerations (see Fig. 4). Most remarkably, only 12.6% of the children adhere for 90% or more of the brushing time of outer surfaces to circular brushing while 30.5% adhere at inner surfaces to vertical brushing for 90% or more of the brushing time. It thus appears as if most children do have considerable problems especially to brush their outer surfaces with circular movements. Still, the majority of children appear to be able to perform these movements as shown by Fig. 4. But they apparently do have severe problems to adhere to them and this might hinder effective toothbrushing education [23]. A simple measure might be to change the sequence of toothbrushing in order to avoid transfer of horizontal toothbrushing from occlusal to lateral surfaces. Starting on the inner surfaces might also increase the awareness of the need to clean these surfaces. Furthermore, as there is little scientific evidence proving an advantage of any of the brushing movements observed here above one other [39, 40], the current results suggest to reconsider brushing recommendations regarding movements given to the children.

A second main finding is that children use dramatically less time for cleaning the inner compared to occlusal or outer surfaces (which they both brushed a similar duration; see Fig. 1). This result is surprising considering that children had been asked to brush their teeth to the best of their abilities and that brushing instructions suggest brushing inner surfaces twice as long as outer surfaces. This is because inner surfaces have to be brushed sextant by sextant while on the outer surfaces two antagonistic sextants can be brushed at a time when children close the mandibles as recommended. Nevertheless, even if children would not adhere to that recommendation one should not expect them to brush outer surfaces so much longer. Actually, the neglect of inner surfaces appears to be the main problem compared to prolonged brushing of outer surfaces. The majority of the children (56.3%) do not manage to brush the inner surfaces of all sextants but miss at least one, twenty-eight children (16%) miss them all (see Fig. 5). This needs further exploration. Potential reasons are decreasing motivation, difficulties in performing the required movements, and incomplete visual control of the tooth brushing. Since the present study did not control any of these potentially influencing factors, it is not possible to decide which of them were responsible for the neglect of the inner surfaces in our participants. However, with respect to motivation the current data argue against that explanation. Children who completely adhere to the recommendations of the brushing song would show a total tooth contact time (without spitting out, changing the position of the brush etc.) of approximately 100 s. The children under study showed a mean tooth contact time twice that long. Thus, the motivation to clean the teeth to the best of their abilities was apparently high. This makes the other factors more probable. Indeed, the additional finding about the distribution of the brushing time to sextants within inner and outer surfaces supports the hypothesis of visual control and/or difficult movements: On the outer surfaces, the children concentrated on the perfect visible front region (longer brushing times at the 2nd and 5th sextant). Concerning the inner surfaces, brushing times indicate that especially the lateral sextants of the maxilla appear to be difficult to reach (see Fig. 2). This is supported by other studies which also found a neglect of posterior inner surfaces in different age groups [35, 41–43]. While in younger children this neglect might be due to missing motor skills [35, 38, 43], children at the age of 12 should have acquired these skills already. Nonetheless, it might be less comfortable to brush these surfaces or one might assume that the visibility of plaque plays a major role when it comes to the decision of where to brush.

Summarizing, the present analysis indicates that children appear to have severe difficulties to adopt the tooth-brushing recommendations provided in prevention programs. This is surprising, as the programs take into account children’s level of development. Furthermore, great endeavors are made to help children internalize the recommendations. A brushing song with a simple text and a catchy melody supports the training. The brushing song is widely distributed to encourage teachers and parents to play it regularly while the children brush their teeth. This should help children to make the application of the recommendations a firm habit, thereby making high quality toothbrushing an automatic well-trained behavior and not requiring further mental efforts [31, 44]. However, the results of the present analysis indicate that these endeavors do not yield the desired results. Children not only appear to have difficulties to apply the requested brushing movements but also do not manage to consider all surfaces of all teeth while brushing. Thus, further research is needed in order to better understand what impedes the adoption of the brushing recommendations. One way of approaching this question is to analyze more closely the significant inter-individual differences observed in this study. Future analyses should explore which factors might contribute to these differences. Family conditions and social variables are key candidates for such analyses [45, 46].

The present study has several strengths such as the large study population, the complex and thorough analysis of brushing behavior, the availability of a standardized instruction procedure that allowed for a differentiated analysis of behavioral adherence and the analysis of children from two cities with different training teams. However, there are also some limitations. First, though children of two cities were included in the analyses, it remains unclear how the results would apply to other regions or even countries. Thus, further analyses are needed in this aspect. Still, to the best of the authors’ knowledge, this is the first study so far to run such a detailed analysis of children’s tooth-brushing behavior and relate it to the recommendations they had been given. Second, even though the intention of the study was to draw random samples of children living in the two cities there was a high portion of children who did not respond to recruitment or denied participation. Thus, the present sample cannot be considered representative of the respective cities. Instead, self-selection might have biased results. One would expect that especially children who feel that their oral hygiene is below average would actively or passively deny participation. Thus, the present results rather overestimate than underestimate children’s toothbrushing abilities. Third, although the instructions given by the brushing song are well standardized and follow common recommendations [31, 44, 47], there is still no scientific evidence supporting these recommendations. Future research should assess which recommendations and training procedures bring about the best results. The analyses of the present study provide important insights for such a research as they already indicate which recommendations may be difficult to adopt for the children.

## Conclusion

In conclusion, evaluations of prevention programs supporting oral hygiene in children with respect to their effects on brushing abilities were missing so far. The present analysis provides such data. Results indicate that brushing abilities of the children remain low despite these programs. This and large inter-individual differences call for future studies analyzing which factors impede adoption of tooth-brushing recommendations in children and which efforts are necessary to improve their toothbrushing abilities.

## Additional file

Additional file 1: Flow diagram of participant recruitment. Shows the complete flow diagram of participant recruitment in Giessen and Marburg. (PPTX 65 kb)

## Abbreviations

ANOVA: Analysis of variance
DMFT-Index: Index of decayed, missing, filled teeth
GAF: Ghassan Al Falouji (assistant)
ICC: Intraclass correlation
LH: Lisa Hassebrauck (author)
OC: Oliver Cordes (author)
SD: standard deviation
