# Supplementary material for: Toothbrushing behavior in children – an observational study of toothbrushing performance in 12 year olds
Source: BMC Oral Health. 2019 Apr 29;19:68. doi: 10.1186/s12903-019-0755-z (PMC6489256; doi:10.1186/s12903-019-0755-z)
Supplement: Supplementary file 1 — Flow diagram of participant recruitment. Shows the complete flow diagram of participant recruitment in Giessen and Marburg. (PPTX 65 kb) [file 12903_2019_755_MOESM1_ESM.pptx]

## Slide 1
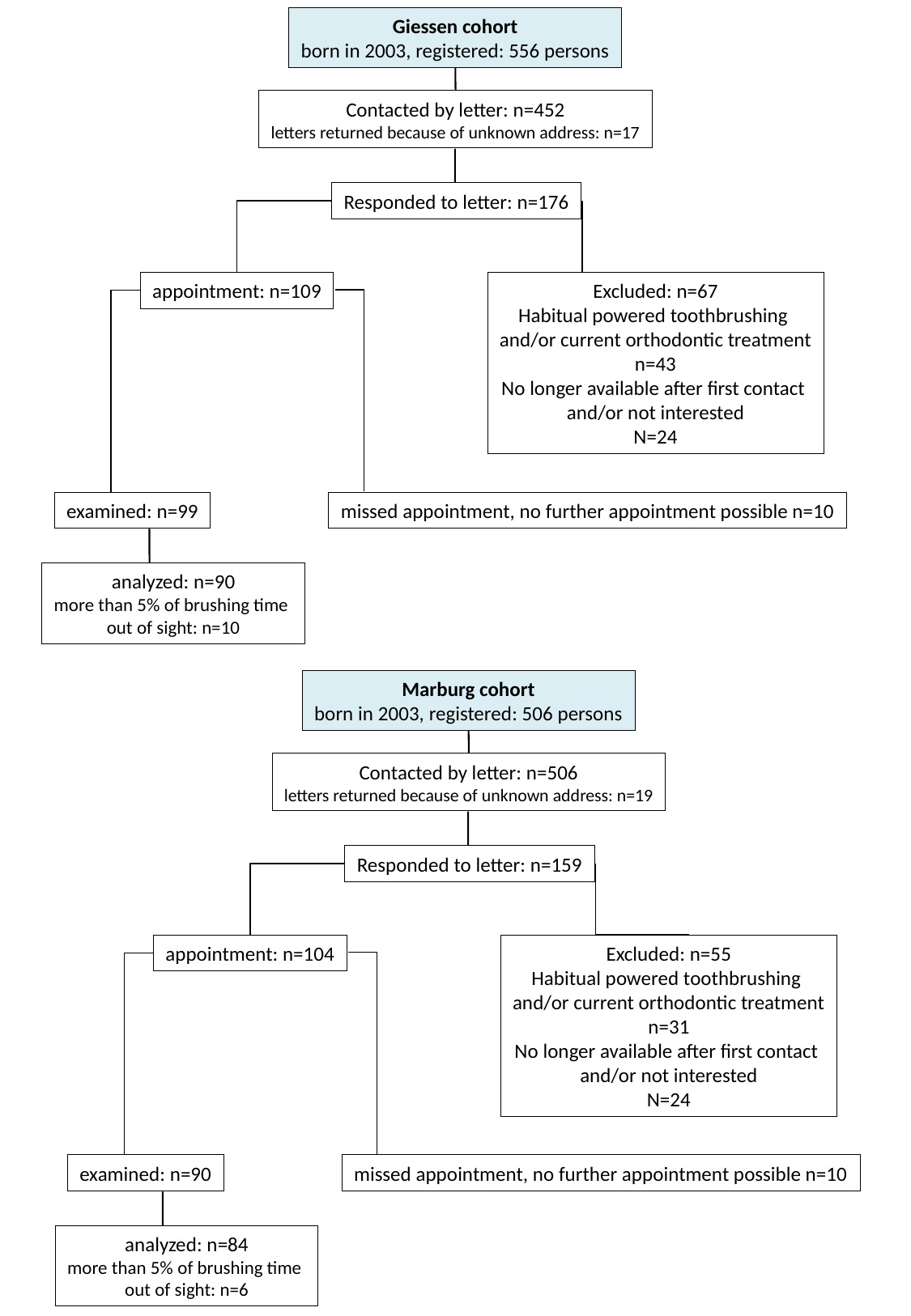

Giessen cohort
born in 2003, registered: 556 persons
Contacted by letter: n=452
letters returned because of unknown address: n=17
Responded to letter: n=176
appointment: n=109
Excluded: n=67
Habitual powered toothbrushing
and/or current orthodontic treatment
n=43
No longer available after first contact
and/or not interested
N=24
examined: n=99
missed appointment, no further appointment possible n=10
analyzed: n=90
more than 5% of brushing time
out of sight: n=10
Marburg cohort
born in 2003, registered: 506 persons
Contacted by letter: n=506
letters returned because of unknown address: n=19
Responded to letter: n=159
appointment: n=104
Excluded: n=55
Habitual powered toothbrushing
and/or current orthodontic treatment
n=31
No longer available after first contact
and/or not interested
N=24
examined: n=90
missed appointment, no further appointment possible n=10
analyzed: n=84
more than 5% of brushing time
out of sight: n=6
